# Monkeys fight more in polluted air

**DOI:** 10.1038/s41598-020-80002-z

**Published:** 2021-01-12

**Authors:** Aichun Xu, Chunhong Liu, Yue Wan, Yali Bai, Zhongqiu Li

**Affiliations:** 1 College of Life Sciences, China Jiliang University, Hangzhou, 301118 China; 2 Lab of Animal Behavior & Conservation, School of Life Sciences, Nanjing University, Nanjing, 210023 China; 3 Lab of Animal Behavior & Cognition, Nanjing Hongshan Forest Zoo, Nanjing, 210023 China

**Keywords:** Ecology, Zoology

## Abstract

Air pollution is a global environmental problem, and its effects on human behavior, psychology, and health have been well studied. However, very few studies were done on if and how air pollution affects animal behavior, for example, social conflict. Many physiological and psychological evidences suggest a possible positive relationship between air pollution and animal social conflict, thus we established a multiple linear regression model using a captive monkey group to explore if monkeys behave more aggressively in polluted air. Our results confirmed that daily social fighting behaviors occurred more when air is polluted. Temperature has a nonlinear effect on monkey social conflict, with a fighting peak at 25–29 °C. To our knowledge, this is the first report that animal social conflict, like humans, is also affected by air pollution and temperature.

## Introduction

Air pollution is a global environmental problem and the situation is much more serious in rapidly developing countries, including China^1,2^, India^3,4^, Mexico^5^. Many studies have documented adverse effects of air pollution on a wide range of outcomes of humans including health, behavior, physiology and psychology in the short- and long-run^6–8^. For example, air pollution harms human health and affects human behavior, in long-run, it can increase the incidences of heart disease, lung cancer, and high blood pressure^7,9^; in short-run, it can impair human cognition, induce avoidance behavior, and cause psychological abnormality^10–12^. Like in humans, air pollution has been reported to cause serious health problems and behavioral responses for animals^13,14^. For instance, the accumulation of heavy metals and fine particles results pigeons in liver and lung damage^15,16^, and pigeons even home faster through polluted air due to the increased perception of predation risk or enhanced navigation ability^17^.

However, whether and how air pollution affects social interactions in humans and animals keeps unknown^18–20^. As we have known, environmental factors, such as temperature and precipitation, especially the former, has an important influence on human social conflict^19,21,22^. Several studies have shown that the global warming might increase civil war or conflict, especially in low income countries^21,23^. Besides the economic reasons, the physiological association linking high temperature and aggression appears robust, although the causal mechanism is not clear^20^.

Similarly to temperature, air pollution might play an important role in shaping social interactions^24^. Physiologically, air pollution might affect central nervous system through at least ultra-fine toxicant or inflammatory response. For example, Ozone is an active substance that can react with molecules in the body to create toxins. It can also trigger an inflammatory response in the central nervous system, and there are many studies documenting that neuroinflammation can trigger increased aggression, impulsivity and depression^25,26^. CO, another important air pollutant, binds to haemoglobin, thus preventing it from accepting oxygen and leading to hypoxia and cognitive impairs^27^. Air pollution might work on social interactions through another path, meaning psychological response. Exposure to air pollution may trigger depress, anxiety, irritation, pain, and discomfort, which may induce aggression^11,12,28^. For example, the incidence of headache of humans is significantly correlated to the ambient exposure to CO and NOx^29^.

Unfortunately, few studies have explored whether and how air pollution affects social conflict of humans^24^, not to mention other animals. If the physiological and psychological mechanisms work similarly as humans, we can predict an aggressive response to air pollution, meaning more fighting behaviors in polluted air. In Nanjing Hongshan Forest Zoo of China, there is an isolated captive group of Rhesus monkey (*Macaca mulatta*). Thus, we video-monitored all the 90 individuals and collected one year round data of social fighting behavior, to explore whether and how air pollution, as well as other environmental factors, affect the social conflict of these monkeys. Our prediction is daily social fighting behaviors would increase as a response to more polluted air and higher temperature.

## Results

The captive monkey group consisted of about 90 individuals, and it was video-monitored by a monitoring camera web. We reviewed all the videos and obtained social conflict data for one year from Mar. 2017 to Feb. 2018. Temperature ranged from − 1 to 40 °C, with an average of 20.8 °C. AQI ranged from 20 to 307, with an average of 76.3. Daily social conflicts ranged from 69 to 258, with an average of 147.1.

**Figure 1:**
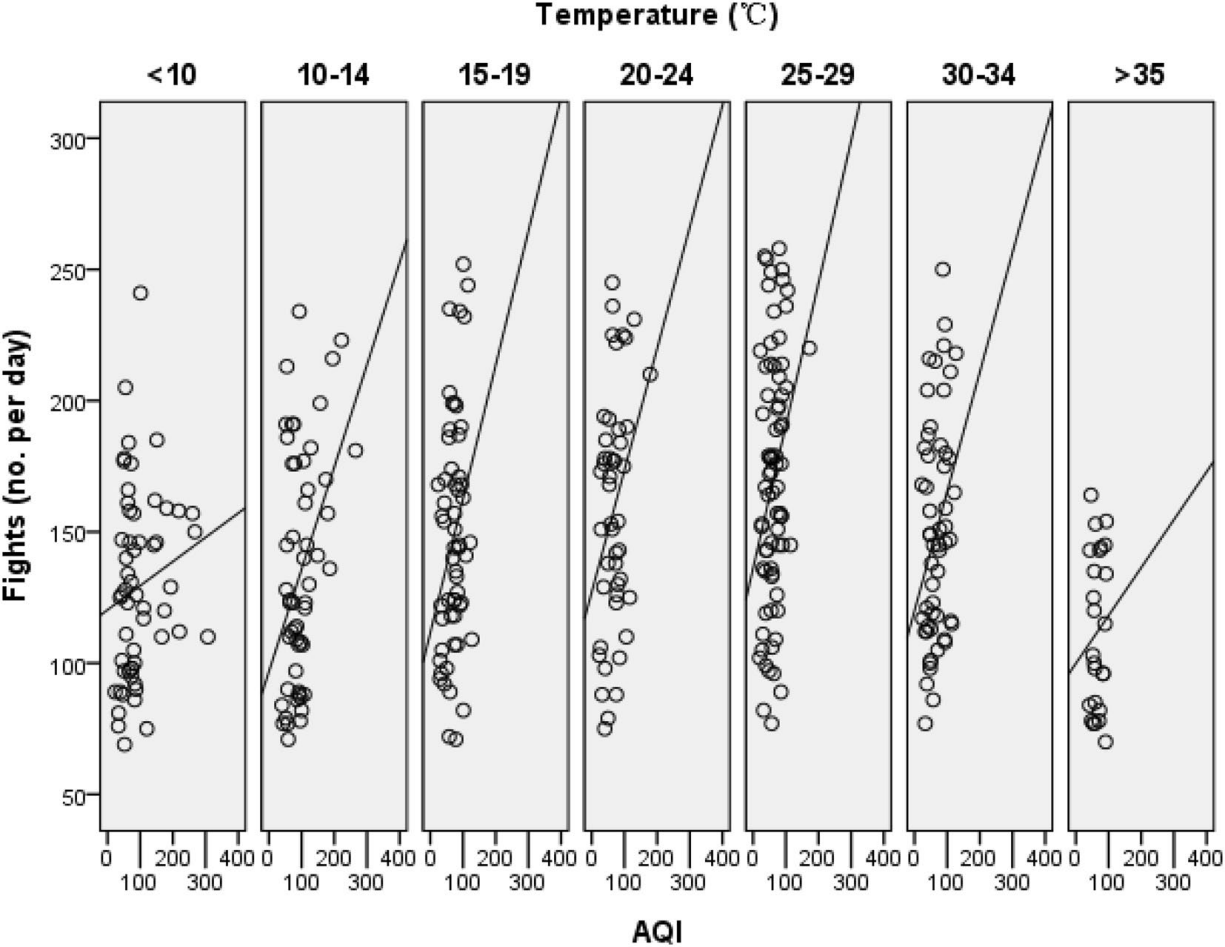
The effect of air quality (AQI) and temperature on social conflict of a captive monkey group in Nanjing Hongshan Zoo in China.

The linear regression model explained 21.3% of the total variance in social conflict with F_10, 347_ = 9.411 (*P* < 0.001, Table 1). AQI had a positive effect on the social conflict of monkeys (F_1, 347_ = 14.020, *P* < 0.0001, Fig. 1). Partial η^2^ of AQI was 0.039, indicating a small effect on social conflict. The temperature had an adverse U-shape effect on the social conflict of monkeys (F_6, 353_ = 9.807, *P* < 0.001, Fig. 1), meaning that the social conflict first increased and then decreased, with a peak (β ± SE = 0.453 ± 0.067, t = 6.749, *P* < 0.001) at 25–29 °C. Partial η^2^ of temperature was 0.145, indicating a large effect on the social conflict of monkeys. Monkeys behaved much more aggressively on days with more visitors (F_1, 347_ = 7.162, *P* = 0.008). Weather had no effect on the social conflict of monkeys (F_2, 347_ = 1.951, *P* = 0.144).

**Table 1:** The regression model exploring air pollution effects on social conflict (Ln) of monkeys in Nanjing Hongshan Zoo in China.

	Estimate	SE	t	*P*	Partial η^2^
Intercept	**4.412**	0.104	42.545	< 0.001	0.839
Visitor	**0.007**	0.003	2.676	0.008	0.020
Weather—sunny or cloudy	0.148	0.089	1.671	0.096	0.008
Weather—overcast or light rain	0.102	0.087	1.170	0.243	0.004
Weather—heavy rain	0^a^	–	–	–	–
Temperature—≤ 10 °C	0.120	0.072	1.654	0.099	0.008
Temperature—10–14 °C	0.121	0.072	1.687	0.093	0.008
Temperature—15–19 °C	**0.249**	0.070	3.554	< 0.001	0.035
Temperature—20–24 °C	**0.320**	0.073	6.124	< 0.001	0.052
Temperature—25–29 °C	**0.420**	0.069	6.124	< 0.001	0.098
Temperature—30–34 °C	**0.272**	0.071	3.841	< 0.001	0.041
Temperature—≥ 35 °C	0^a^	–	–	–	–
AQI	**0.002**	0.000	3.744	< 0.001	0.039

Significant results were marked bold.

^a^This parameter is set to zero because it is redundant.

Compared to the above model, the one day lag regression model explained 12.2% of the total variance in social conflict with F_10, 347_ = 4.830 (*P* < 0.001, Table 2). Temperature with one day lag has a significant effect on social conflict (F_6, 347_ = 3.556, *P* = 0.002), but the effect was lowering to median with Partial η^2^ of 0.058. AQI with one day lag has no effect on social conflict (F_1, 347_ = 2.412, *P* = 0.121), with Partial η^2^ of 0.007.

**Table 2:** The one day lag regression model exploring air pollution effects on social conflict (Ln) of monkeys in Nanjing Hongshan Zoo in China.

	Estimate	SE	t	*P*	Partial η^2^
Intercept	4.573	0.109	42.091	0.000	0.853
Visitor	**0.010**	0.003	3.407	0.001	0.032
Weather—sunny or cloudy	0.139	0.092	1.512	0.131	0.007
Weather—overcast or light rain	0.104	0.091	1.141	0.255	0.004
Weather—heavy rain	0^a^	–	–	–	–
Temperature 1 day lag—≤ 10 °C	0.065	0.076	0.847	0.398	0.002
Temperature 1 day lag—10–14 °C	0.073	0.075	0.969	0.333	0.003
Temperature 1 day lag—15–19 °C	0.095	0.074	1.287	0.199	0.005
Temperature 1 day lag—20–24 °C	**0.229**	0.077	2.974	0.003	0.025
Temperature 1 day lag—25–29 °C	**0.239**	0.071	3.343	0.001	0.031
Temperature 1 day lag—30–34 °C	**0.162**	0.074	2.177	0.030	0.013
Temperature 1 day lag—≥ 35 °C	0^a^	–	–	–	–
AQI 1 day lag	0.001	0.000	1.553	0.121	0.007

^a^This parameter is set to zero because it is redundant.

## Discussion

Aggression or social fighting behavior is one of the basic instincts and its origin has become a central theme in debates about human social evolution^30^. There are many studies on social conflicts in primates, but basically nearly all focus on the types, causes and functions^31^. The causes why animals fight with each other mainly come from the direct competition for foods, mates, territories or social rank^30^. Thus for an isolated social group, the generation of aggression comes more from the internal causes. However, the external or environmental factors undoubtedly affect the behavioral expression of primates through physiological and psychological pathways, and then induce or inhibit social conflict. This may explain why our model only explains 21.3% of the variation, because we only focused on the environmental factors, and did not consider the internal factors that induced the aggressions.

Our regression model revealed that air pollution probably plays an important role in shaping social conflicts in monkeys, even though the effect size is small. Many medical, physiological and economic literatures suggest two pathways by which air pollution might affect human behavior: a physiological one, through which air pollution directly affects the physical and cognitive function of the central nervous system; and a psychological one, through which air pollution causes irritation and discomfort which itself induces aggressive behavior^25,26,28,32^. However, we don’t know if air pollution affects animal social conflict, and if so, what is the pathway? A recent paper found that social status changes immune cell gene expression and chromatin accessibility through glucocorticoid regulation, thus leading to disease susceptibility in monkeys with low social rank^33^. Another paper reviewed the association between air pollution and glucocorticoid responsiveness, and tried to explore the underlying mechanisms that how air pollution affects glucocorticoids, including changes to phosphorylation or oxidation of the glucocorticoid receptor, repression by cytokines, or inflammatory pathways, and epigenetic effects^34^. Many other studies also provided enough evidences that air pollution may induce respiratory functional impairment^13,35^, or brain development problems in monkeys^36^. All above indicated that air pollution can affect monkey social aggression, at least by physiological pathway^32^. However, to our knowledge, we don’t know if the other psychological pathway work. We know that in many animal welfare studies, when monkeys living in a narrow space or monotonous environment move to an open area or enriched environment, their stereotypical behaviors that are believed to indicate decreased psychologic well-being decreased as expected^37–39^. This means environmental factors probably can affect monkeys’ psychological status, but more studies are needed.

Besides air pollution, temperature might be a more important factor shaping the fighting behavior patterns of monkeys. To our knowledge, traditional field studies on animal fighting behavior focus primarily on the evolutional origin, function or fitness of social conflict^31^, very few take consideration of environmental impact. However, lots of studies on human conflict, across spatial scales from a single building to the globe and at temporal scales ranging from an hour to a millennium, have shown an apparent relationship between social conflict and the temperature^20,21^. Though some studies assume a linear relationship between temperature and social conflict, more evidence suggests that temperature appears to have a nonlinear relationship with conflict over a large range of temperatures^20,40^. Our results also suggest a nonlinear relationship, the fighting behaviors firstly increase and then decrease, with a conflict peak at 25–29 °C. Monkeys behave less aggressively when temperatures are lower than 15 °C or higher than 35 °C. Several causal mechanisms or hypotheses have been proposed to explain the temperature effect on social conflict in humans^20^. Instrumental variables from social science aspect, like economic factors, are usually used to link human social conflict and temperature or other climate factors^20^, however, since our objectives are captive monkeys with enough daily food supply, and they don’t even have to consider any dietary shortage (which might be similar to economic reason in humans), thus this potential influence should be omitted. As suggested above, physiological or psychological pathways might work, but further studies are needed.

Other factors, especially daily visitor numbers have an influence on fighting behaviors, monkeys behave more aggressively when there are more zoo visitors. Visitor effect include unruly behaviors of visitors such as teasing, feeding, shouting and even throwing stones at captive animals, and even just visitor presence sometimes can lead to behavioral, physiological and psychological changes in these animals^41^. Particularly, the aggressive behaviors of these primates increase with the number of visitors^42,43^. There are usually much more zoo visitors at weekends and on sunny days^44,45^, thus probably leading to an unexpected visitor effect on the social interactions of these monkeys. One day lag regression model indicates that not air pollution one day before but the temperature one day before has a medium effect on social interactions of these monkeys. This indicated that air pollution has an immediate and short-lived impact on animal conflict.

In conclusion, many scientists have studied how environmental factors especially the global climate change affect human social conflict, from both temporal and spatial scales. However, to our knowledge, this is the first case report linking animal social conflict and environmental factors. Air pollution, as well as global warming, are likely to have effects beyond imagination, both for humans and animals.

## Methods

### Monkey conflict data

We obtained social conflict data ofNorthern China Rhesus Monkeys from Hongshan Forest Zoo of Nanjing, China. Nanjing (31° 14′–32° 37′ N, 118° 22′–119° 14′ E) is located in the central region of the lower Yangtze River and southwest of Jiangsu Province. It is an important national gateway city for the development of the central and western regions in the Yangtze River Delta, with an area of 6587 km^2^ covering a population of more than 8 Million. Average annual temperature is about 15.4 °C. Annual precipitation is 1106 mm, 60% of which occurs from Jun to Sep.

There are about 90 monkeys in the Hongshan Zoo in 2017, about 35 adults, 20 sub-adults and 35 juveniles or new-borns. The round monkey park was located in the central part of the zoo, with an area of about 2000 m^2^. Although a thick and 3-m high glass wall has been built to prevent artificial feedings, visitors sometimes throw food into the monkey park, causing a social conflict due to the food competition. Usually the zookeeper feeds these monkeys twice a day at about 9:30 am and 3:30 pm respectively.

We established a monitoring camera web (Haikang DS-7104N-SN/P) covering the monkey park in September 2016 and video-recorded the whole population since then. We defined social conflicts of monkeys as aggressive or fighting behaviors between individuals, including chasing (one chases another until it escapes), wrestling (one grapples and wrestles with another, until one escapes or gives up), biting (one opens its mouth and bites or tries to bites another), scratching (One scratches or scrapes another using its hands), threating (One warns or threats another through calling or behavioural display), etc. The age of participants and the occurrence time were recorded for each aggression^46^. We considered a conflict ends if there is no continuation within 10 s after the aggression. Since these monkeys are inactive during the night, we only recorded their diurnal aggressive behaviors from 6:30 till 18:30 and then summed the fights as daily social conflicts. One-year round data were collected from Mar 2017 to Feb 2018.

### Air Quality Index

We obtained Air Quality Index (AQI) data of Nanjing from the Data Centre of the Ministry of Environmental Protection of the People’s Republic of China (MEP, http://datacenter.mep.gov.cn/)^17^. Based on established criteria (GB3095-2012). AQI is calculated for six major air pollutants separately: particle matter < 10 microns in diameter (PM10, μg/m^3^), particle matter < 2.5 microns in diameter (PM2.5, μg/m^3^), ground-level ozone (O_3_, μg/m^3^) level, carbon monoxide (CO, mg/m^3^) level, sulphur dioxide (SO_2_, μg/m^3^) level, and nitrogen dioxide (NO_2_, μg/m^3^) level. An individual score is assigned to the level of each pollutant and the final AQI is the highest of those 6 scores. AQI values range from 0 to 500, and can be classified into six categories (Good: 0–50, Moderate: 51–100, Unhealthy for Sensitive Groups: 101–150, Unhealthy: 151–200, Very Unhealthy: 200–300, Hazardous: 301–500).

### Meteorological variables

We obtained meteorological data from a public weather website (http://www.tianqihoubao.com/). We collated data of weather conditions, wind force and ground air temperature (°C). Based on these data, we defined the weather conditions as: sunny or cloudy; light rain or overcast; and heavy rain or snow. Wind force was classified into two categories: breezy or windy. According to the sample distribution, we defined breezy as wind force less than 3rd level (China wind level standard, wind speed less than 5.4 m per sec), and windy as wind force more than 3rd level. We categorized temperature into 7 levels: < 10 °C, 10–14 °C, 15–19 °C, 20–24 °C, 25–29 °C, 30–34 °C, and ≥ 35 °C. We did not deal temperature as a continuous variable since many similar studies in social science categorized the temperature^24,47^. To simplify the possible effect of week days, we categorized the dates into two types: weekdays, and weekends or statutory holidays (including Spring Festival, Ching Ming Festival, Labor Day, Dragon Boat Festival, Mid-Autumn Festival, National Day, New Year's Day).

### Data analysis

Many social and environmental factors can affect social conflicts^18,19,22,31^, but for a given population like Hongshan monkeys, social conflict can be considered as an independent variable to a certain extent that responds to the real-time environmental factors. There might be an effect of time series on the occurrence of social conflict^18,24^, such as day of week, date of month, statutory holidays However, this time series factor may play a role in studies of human behavior, psychology or sociology, but not in other animals, because obviously other animals did not have the time conception of humans, and should not be affected by this time factor. But they can feel if there are more visitors (and therefore more supplemental foods and more human disturbance) on holidays. We actually found a positive correlation between the time factor (weekday/holiday) and daily visitor numbers (r > 0.6), thus we replaced time factor with daily visitor numbers. So in our general linear model, we included 5 independent variables: weather (Sunny or Cloudy, Overcast or Light Rain, Heavy Rain or Snow), wind force (Breezy that wind speed less than 5.4 m per sec, Windy that wind speed more than 5.4 m per sec), temperature (7 levels (< 10 °C, 10–14 °C, 15–19 °C, 20–24 °C, 25–29 °C, 30–34 °C, ≥ 35 °C), air quality indexes (Continuous variable) and visitor numbers (Continuous variable). Another thing that we were caring was that the fighting behavior may be affected by the environmental factors, especially the temperature and AQI that happening prior to the fight occurring, so we established a one day lag regression model including the weather, wind force, holidays, temperature 1 day lag, and AQI 1 day lag as fixed factors. We did not find a significant collinearity among these factors (VIF < 2), and so we kept all the factors in the models. We also tried the models using temperature as a continuous variable and even with a quadratic function of temperature, but these models explained much less variance (less than 14%) than our current models, so we decided using categorized temperature in our final analysis. Wind force has no effect in both models (*P* > 0.9), so we removed the wind force from the final models.

We calculated partial η^2^ to identify the relative importance of each factor after controlling for other factors. As suggested, values of partial η^2^ with 0.01, 0.06 and 0.14 indicate small, medium, or large effects for any measure of the proportion of variance explained^48,49^. All analyses were conducted with SPSS 18.0, significant level was set at 0.05, and statistical values were shown as mean ± SE.

### Ethic information

This is an observational study, we did not take any measures on these animals except video recordings.
